# Physiochemical Properties of Experimental Nano-hybrid MTA

**Published:** 2018

**Authors:** Vagharedin Akhavan Zanjani, Kasra Tabari, Seyedeh Mahsa Sheikh-Al-Eslamian, Ahmad Najafi Abrandabadi

**Affiliations:** * Department of Restorative Dentistry, Dental School, Shahid Beheshti University of Medical Sciences, Tehran, Iran; ** Dental Research Center, Research Institute of Dental Sciences, Dental School, Shahid Beheshti University of Medical Sciences, Tehran, Iran

**Keywords:** Acidity, Calcium, Ions, Mineral trioxide aggregate, Nanoparticles

## Abstract

**Introduction:**The development of new pulp capping agents has paved the way towards the preservation of pulp vitality, which is an important goal in restorative dentistry. This study sought to assess the calcium ion release, pH and setting of mineral trioxide aggregate (MTA) Angelus, an experimental formulation of nano-hybrid MTA containing nano-SiO2, nano-Al2O3 and nano-TiO2 and MTA Angelus plus nano-oxides.

**Methods:**In this experimental study, five specimens from each material were placed in polypropylene tubes and immersed in a flask containing deionized distilled water. The amount of calcium ions released into the solution from each material was measured at 15 minutes, one hour and 24 hours using atomic absorption spectroscopy. The pH of the solutions was measured using a pH meter at the respective time points. Setting time was also assessed using a Gilmore needle. Data were analyzed using the repeated measures ANOVA.

**Results:**The amount of released calcium ions was not significantly different among the groups (P=0.060). All materials were alkaline and the pH at 24 hours was significantly higher than the other two time points in all groups (P<0.001). The experimental group had the shortest and MTA Angelus had the longest setting time. All materials were alkaline and capable of releasing calcium. Addition of nanoparticles to MTA Angelus significantly decreased the setting time but had no effect on the release of calcium ions or pH.

**Abbreviations:** mineral trioxide aggregate (MTA), vital pulp therapy (VPT)

## Introduction

The development of new pulp capping agents has paved the way towards the preservation of pulp vitality, which is an important goal in restorative dentistry. The purpose of vital pulp therapy (VPT) is maintenance and stimulation of the remaining healthy tissue to promote healing of the dentin-pulp complex. Suitable candidates for VPT include vital primary/permanent teeth with full/partially root formation after traumatic/carious/iatrogenic pulp exposure. In addition, adequate blood supply, severity of inflammation, hemostasis, disinfection of exposure/cavity preparation and coronal seal are important criteria for successful treatment [**1**][**2**].

Nanotechnology has enabled the production of nanoscale dental materials with improved physicochemical properties. Different nanoscale oxides such as SiO2, TiO2 and Al2O3 nanoparticles have been incorporated into the formulations of dental materials and cement to enhance their physical and mechanical properties and long-term clinical success [**3**][**4**]. These nanoparticles provide greater surface area and higher reactivity and excitability [**4**]. Addition of nano-Al2O3 to cement increases the compressive strength and modulus of cement elasticity. Incorporation of nanoparticles fills the gaps and creates a less porous, dense mass leading to an increase in the modulus of elasticity [**5**]. Addition of 3wt% nano-SiO2 to cement was shown to increase the reactivity and 7-day and 28-day compressive strength and decrease the size of Portland crystals [**6**]. Nano-TiO2 is also used due to its unique anti-bacterial, self-cleaning and photoelastic properties.

MTA (Mineral Trioxide Aggregate) is composed of tricalcium silicate, dicalcium silicate, tricalcium aluminate, tetracalcium aluminoferrite, calcium sulfate and bismuth oxide and is available in two forms of white and gray MTA. It has unique properties and provides an optimal seal, which is improved over time. It was first used for the apical seal in retrograde surgeries but at present it has numerous applications such as repair of perforations in the pulp chamber floor, repair of furcal perforations, induction of the formation of an apical barrier in immature necrotic teeth and pulp capping in permanent teeth.

Nano-hybrid MTA is a newly invented cement with Portland based cement that contains nano-SiO2, nano-Al2O3, nano-TiO2 and microsilica for more favorable physicochemical properties and Bi2O3 for greater radiopacity. The experimental cement was fabricated in the laboratory of the Parseh Dental Promotion Center (Tehran,Iran) by Kasra Tabari [**6**]. The manufacturer claims that this cement has optimal properties. However, further studies are required to test the efficacy of this cement.

To induce mineralization, a material must have high pH and should be capable of releasing calcium ions. These capabilities are required for hard tissue healing [**7**]. Calcium ions released from the cement react with phosphate ions present in body fluids and lead to mineralization and formation of hard tissue. MTA was shown to produce deposits resembling hydroxyapatite in both composition and structure in synthetic tissue fluids [**8**]. A high pH is also a requisite for pulp capping agents since high pH is directly correlated with antibacterial cement activity, and is critical for the formation of a hard tissue barrier [**9**].

Although the release of calcium ions and the pH of MTA have been the subject of many previous investigations, the comparison with nano-hybrid MTA has not been studied [**10**][**11**]. Moreover, finding a solution to shorten the setting time of these materials without changing their pattern of calcium ion release while creating an alkaline environment will eliminate a major obstacle against the current use of these materials in the clinical setting.

Considering the need to overcome the shortcomings of the commercially available MTA, this study sought to assess the calcium ion release, pH and setting time of MTA Angelus, MTA Angelus plus nano-oxides and an experimental formulation of nano-hybrid MTA.

## Materials and methods

The pH, setting time and calcium ion release of MTA Angelus (Angelus, Londrina, PR, Brazil), MTA Angelus plus nano-oxides and an experimental formulation of nano-hybrid MTA were evaluated in this in-vitro experimental study. MTT analysis was previously used to show the biocompatibility of nanoparticles and experimental nano-hybrid MTA [**12**][**13**]. A total of 5 specimens were fabricated from each cement with the following compositions:

- Experimental nano-hybrid MTA: Portland cement + Bi2O3 + nano-silica + nano alumina + nano-TiO2 + microsilica + nano-hydroxyapatite (powder/liquid ratio: 3/1) - MTA Angelus (powder/liquid ratio: 3/1)

Polypropylene tubes with an internal diameter of 1 mm and a height of 1 cm were used for this purpose. The tubes were weighed before and after filling them with cement in order for all tubes to have the same weight of cement material. All cement inside the tubes weighed approximately 0.05 g. The pastes were packed inside polypropylene tubes using a hand condenser. The excess water at the two ends of the tube was absorbed by a paper towel, and cement residues were cleaned from the external walls. To reduce error, five specimens from each cement were fabricated. The specimens were then gently transferred into glass flasks containing 10 mL of deionized distilled water and the lids were sealed. To prevent sample contamination, glass flasks were washed with 3M nitric acid, thoroughly rinsed with deionized distilled water and dried in an oven before use. The flasks containing the specimens were incubated at 37°C with >90% relative humidity. To measure the amount of calcium ions released and pH at 15 minutes and one hour, the five specimens made from each cement were carefully transferred to another flask containing fresh deionized distilled water and after measurement of the pH and the total calcium ions released into the solution, the specimens were returned to the primary flask. At 24 hours, the measurements were repeated, but the specimens were put aside and were not returned to the first flask. The solution inside the flasks was tested to measure the pH and the amount of calcium ions released. For the pH measurement, the pH meter (Model 744, Metrohm, Switzerland) was first calibrated using standard solutions with pH values of 4 and 7 and the probe of the device after each time of use was thoroughly washed with deionized distilled water and dried with paper towel. The pH measurements were made at 28±1°C.

To measure the quantity of calcium ions released, atomic absorption spectroscopy (Perkin-Elmer model 1100B, Phoenix, AZ, USA) was used (air/acetylene flame, calcium cathode lamp). First, a standard calibration curve was drawn using standard calcium chloride solutions containing 1, 2, 3, 4 and 5 ppm calcium concentrations. Next, 20μL of 5% lanthanum chloride solution was added to 1 mL of each standard solution, and the standard samples were injected into the device. The lanthanum chloride solution prevents other ions, especially phosphate ions, from interfering with the measurements. To measure the amount of released calcium ions, 100μL of the solution was added to 900μL of deionized distilled water in a test tube; 20μL of lanthanum chloride solution was also added, and the samples were injected into the device. The value displayed for each specimen was recorded. The results were calculated with ×10 correction factor [**14**].

The setting time was defined as the duration of time from the mixing of the material until no indentation could be made on the specimen surface. A Gilmore needle weighing 456.5g was used for indentation to obtain the final setting time. For the setting test, according to ADA specification no. 57 and ASTM specification C266-03, the mixtures were transferred to ring molds measuring 10 mm in diameter and 2 mm in thickness. Three specimens were fabricated in each cement group. After mixing the materials according to the manufacturer’s instructions, a chronometer (Akai, China) with an accuracy of 0.01 second was adjusted to zero and after the mixing time had elapsed, the chronometer was started. After packing the specimens into molds, the surface was smoothed using a metal spatula. The tip of the device (a needle measuring 2 mm in diameter and 4 mm in length) was placed on the horizontal surface of the material for 5-seconds at 30-second intervals. The indentation caused by the tip of the Gilmore needle was an indicative of unset material.

Repeated measures ANOVA were applied to compare the pH and release of calcium ions over time in different groups. To compare the setting time of different groups or pairwise combinations of groups, One-way ANOVA and Tukey’s HSD test were used, respectively.

Type 1 error was considered as α=0.05 and P<0.05 was considered statistically significant.

## Results

**Table 1** shows the mean amount of calcium ions released from the cement. Repeated measures ANOVA revealed a significant increase in calcium ions release over time (P<0.001). However, no significant difference was noted between the 15 minutes and 1 hour time points (P=0.98). However, the release of calcium ions at 24 hours was significantly greater than at the other two time points (P<0.001). The amount of released calcium ions was not significantly different among the cement groups (P=0.060). Moreover, the interaction effect of the group and time was not significant (P=0.315).

**Table 1:** Mean and standard error of calcium ion release and pH in different groups

Groups	Ca ions release(ppm) ±SE			pH±SE		
	15 min	1h	24h	15min	1h	24h
Nano-hybrid MTA	13.80±0.92	12.80±0.90	24.80±0.86	9.47±0.15	9.94±0.12	10.41±0.10
Angelus+nanooxides	10.60±0.92	10.00±0.90	25.20±0.86	9.68±0.15	9.58±0.12	10.58±0.10
Angelus	10.60±0.92	11.40±0.90	24.80±0.86	9.45±0.15	9.63±0.12	10.48±0.10


The mean pH of materials is shown in **Table 1**. Repeated measures ANOVA revealed a significant increase in pH over time (P<0.001). Although the pH was not significantly different at 15 minutes and one hour (P=0.392), the pH at 24 hours was significantly different from that at the other two time points (P<0.001). In this study, the pH was not significantly different among the groups (P=0.564). The interaction effect of group and time was not significant either (P=0.315).

The mean and standard error (SE) of the final setting time in different cement groups are shown in **Table 2**. One-way ANOVA revealed a significant difference in this regard among the 3 groups (P<0.001).

**Table 2:** Mean and standard error of final setting time in different groups

Groups	Final setting time (sec)
Nano-hybrid MTA	14.66±1.45
Angelus+nanooxides	24.33±0.88
Angelus	39.00±2.08

Pairwise comparison of groups using Tukey’s HSD test revealed a significant difference between the experimental group and MTA Angelus (P=0.001), but the difference between MTA Angelus + nano-oxides and MTA Angelus was not significant (P=0.27) in this regard. The experimental group had the shortest, and MTA Angelus had the longest setting time.

## Discussion

MTA has numerous applications in direct pulp capping, root repair and furcal perforations and apexification. The MTA powder is mixed with sterile water and a moist cotton pellet is placed directly over the applied restoration since the moisture helps to set the material. Following water sorption, a colloidal gel is formed. The properties of this gel are influenced by factors such as the powder/liquid ratio, the mixing method and the amount of air trapped in the mixture, the pressure applied for condensation, the environmental moisture, the type of MTA, the pH of the environment, the type of liquid mixed with the powder, the thickness of the material and temperature [**15**][**16**][**17**]. Gray MTA has a longer initial and final setting time than white MTA [**18**][**19**]. Longer setting time of white MTA compared to the Portland cement is due to the lower sulfur and tricalcium aluminate contents of white MTA [**20**]. Considering the importance of calcium ion release in hard tissue formation and also the necessity of high pH for antibacterial activity, we aimed to assess the calcium ion release and pH of the experimental formulations of nano-hybrid MTA in comparison with MTA Angelus and MTA Angelus + nano-oxides.

The nano-hybrid MTA contains Portland cement, Ca(OH)2, Bi2O3, nano-SiO2, nano-Al2O3, nano-TiO2, nano-hydroxyapatite and microsilica in different weight percentages. The mechanism of action of Ca(OH)2 is directly based on the solubility of calcium and hydroxyl ions and subsequent increase in pH [**21**]. In addition to the creation of an alkaline environment, which has antibacterial properties, hydroxyl ions are oxidizing free radicals with very high reactivity. They react with different types of biomolecules such as bacterial fatty acids. They inactivate endotoxins and denature proteins in the root canals. By DNA damage, they disintegrate the cytoplasmic membrane of bacteria [**22**]. Calcium hydroxide can enhance repair and calcification of adjacent areas by the distribution of calcium and hydroxyl ions [**23**][**24**]. Nano-hydroxyapatite particles have optimal biological properties, and by adding them to the restorative materials we can benefit from their favorable properties.

Several methods are used for the measurement of released calcium ions such as gravimetry, reverse titration and polarography. However, we used atomic absorption spectroscopy with a cathodic lamp because, in contrast to gravimetry and reverse titration, this method can measure small amounts of ions. Atomic absorption spectroscopy has high measurement accuracy and is among the most widely used techniques for measuring the concentration of elements in solutions [**25**]. Polarography is comparable with atomic absorption spectroscopy in terms of sensitivity; however, the former is time-consuming and complex. Similar studies have also used atomic absorption spectroscopy for measurement of calcium ions released from MTA [**26**].

The amount of released ions was measured at 15 minutes, one hour and 24 hours. The measurement at 15 minutes has not been done in any previous study. Moreover, ion release was not evaluated cumulatively in our study because long-term storage of specimens in the solution leads to a reduction in the release of new ions because the solution becomes saturated with the ions. The release of calcium ions in solutions is based on diffusion due to the passive transfer of ions despite the low solubility of the compound. When MTA is mixed with water, it forms calcium oxide, which releases hydroxyl and calcium ions. In the current study, the release of calcium ions increased over time in all groups and the difference in this regard between one hour and 24-hour time points was statistically significant. This finding shows that release of calcium ions continued throughout the study in all groups. The results of the current study are in line with the findings of Massi et al. regarding the release of calcium ions from the MTA Angelus at 24 hours [**27**].

A pH meter was used for the measurement of pH, which is also in accordance with previous studies [**28**]. The release of hydroxyl ions is based on diffusion. Generally, all groups in our study had an alkaline pH similar to the findings of previous studies [**19**][**21**]. Thus, the possibility of inducing the formation of strong tissues existed in all groups [**28**]. The hydroxyl ions affect the bacterial cytoplasmic membrane, which is necessary for cell metabolism, growth and division [**29**]. The assessment of pH changes in all groups revealed an increasing trend over time. In all groups, the difference in pH was significant at 24 hours compared to the other two time points (15 minutes and one hour). The pH obtained for MTA Angelus in the study by Duarte et al. was lower than the values obtained in our study [**22**]. Although the method of measurement was similar to that employed in the current study, some factors cannot be easily controlled and it is difficult to standardize the MTA properties measurement method. In another study by Torabinejad et al., the pH of MTA Angelus reached 12 within three hours. In their study, the samples were fabricated in the form of discs and thus, a greater surface of the material was in contact with water [**28**]. Our findings were in line with the results of Amini Ghazvini et al., and two studies were also similar in terms of study design and methodology [**14**].

Cement setting time is important since the optimal properties of materials are obtained after the setting time has elapsed. According to the EN 196-3 (2005) standard, a minimum amount of water must be used for mixing with the powder. On the other hand, the amount of water must be adequate for complete hydration. In the current study, as described in other studies [**30**], a Gilmore needle with 456.5 g weight was used to calculate the final setting time and mixing was done by the same operator for all specimens.

Several factors affect the setting time of MTA such as the amount of water used for mixing, the mixing process, the load applied for cement packing, the environmental moisture and temperature. The short setting time of cement decreases the risk of washout and separating MTA [**31**]. Much effort has been made to decrease the setting time such as the addition of calcium chloride [**32**], polymers [**19**] and plasticizers [**33**].

In contrast to the ISO 6876:2001 standard for testing the setting of dental cement that recommends the use of gypsum molds, metal molds with standard dimensions were used in the current study due to the interference of gypsum with MTA setting; this is in accord with previous studies by Torabinejad et al. and Islam et al. [**26**][**28**]. MTA Angelus is a Brazilian cement that sets within 15 minutes according to the manufacturer. Based on the standard conditions of the study, our results (39.00±2.08 minutes setting time) were not in accordance with the manufacturer’s claim in this regard and had a significant difference. However, our findings in this respect were similar to those of Islam et al. and Massi et al. [**26**][**27**].

Addition of nanoparticles to MTA Angelus significantly decreased the setting time in our study. This difference may be attributed to the greater surface area in the group containing nanoparticles. The increased surface area expedites the reaction of the powder and the liquid. Results of the current study are in line with those of Saghiri et al.; the only difference was that in their study, different elements were used to produce the nano white MTA [**31**]. In our study, experimental groups containing nanoparticles yielded different results due to differences in the distribution of elements and their composition. The shortest setting time belonged to the nano-hybrid MTA group, which may be due to the greater hydrophilicity of the Portland cement.

Future studies are required to measure the pH and assess the release of calcium ions over longer periods of time to find out how long this process lasts. Also, for the measurement of ion release, phosphate buffer must be used to better simulate in vivo conditions and assess the behavior of cement in conditions closer to the clinical setting. Addition of fluorapatite instead of hydroxyapatite to the MTA formulation should be investigated as well for possible applications in indirect pulp capping due to the fluoride release potential and the induction of mineralization.

Within the limitations of this study, we found that the studied materials were alkaline and capable of releasing calcium. Changes in the composition of these materials did not affect the release of calcium ions or the pH. The nano-hybrid MTA group showed properties comparable to those of MTA Angelus. The setting time claimed by the manufacturer of MTA Angelus was lower than the obtained results. Addition of nanoparticles to MTA Angelus significantly decreased the setting time but did not affect the release of calcium ions or pH.

**Acknowledgment**

The authors wish to thank the Research Institute of Dental Sciences, Shahid Beheshti University of Medical Sciences, Tehran, Iran.

**Disclosures**

The authors declare that there is no conflict of interest.
